# Supplementary material for: Cost-effectiveness of stem cell therapy versus standard of care for acute and subacute ischemic stroke
Source: PLoS One. 2026 May 21;21(5):e0349756. doi: 10.1371/journal.pone.0349756 (PMC13193451; doi:10.1371/journal.pone.0349756)
Supplement: S1 File — (DOCX) [file pone.0349756.s001.docx]

**Supplemental material**


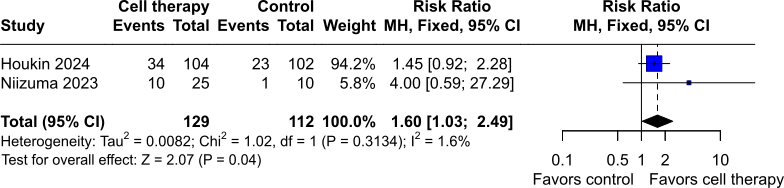


S1 Figure. Forest plot comparing the risks of modified Rankin Scale scores of 0–2 by 90 days after treatment in scenario 2


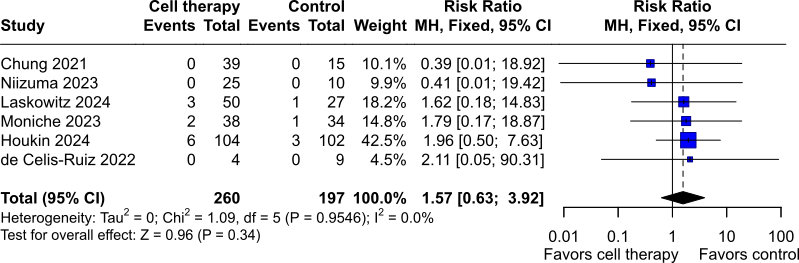


S2 Figure. Forest plot comparing the risks of mortality at 90 days after treatment (main analysis)


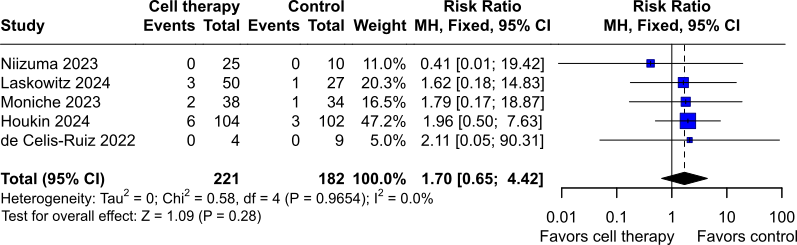


S3 Figure. Forest plot comparing the risks of mortality at 90 days after treatment (scenario 1)


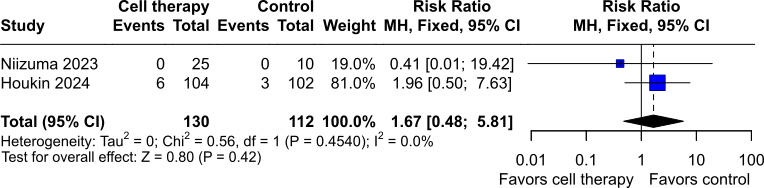


S4 Figure. Forest plot comparing the risks of mortality at 90 days after treatment (scenario 2)

S1 Table. Monthly transition probabilities for Markov model

| Transition | Probability per cycle per month |
| --- | --- |
| Functional independence to disability (mRS score of 0–2 to mRS score of 3–5) | |
| 4–6 months | 0.0319 |
| 7–12 months | 0.0235 |
| 13–120 months | 0.0105 |
| Disability to functional independence (mRS score of 3–5 to mRS score of 0–2) | |
| 4–6 months | 0.0372 |
| 7–12 months | 0.0156 |
| 13–120 months | 0 |
| Functional independence to death (mRS score of 0–2 to mRS score of 6) | |
| 4–12 months | 0.00701 |
| 13–24 months | 0.00373 |
| 25–36 months | 0.00420 |
| 37–48 months | 0.00475 |
| 49–60 months | 0.00538 |
| 61–72 months | 0.00609 |
| 73–84 months | 0.00690 |
| 85–96 months | 0.00783 |
| 97–108 months | 0.00889 |
| 109–120 months | 0.01007 |
| Disability to death (mRS score of 3–5 to mRS score of 6) | |
| 4–12 months | 0.02273 |
| 13–24 months | 0.00872 |
| 25–36 months | 0.00982 |
| 37–48 months | 0.01111 |
| 49–60 months | 0.01259 |
| 61–72 months | 0.01424 |
| 73–84 months | 0.01613 |
| 85–96 months | 0.01831 |
| 97–108 months | 0.02078 |
| 109–120 months | 0.02354 |

This table was modified with permission from that in Morii et al., 2023 [9], licensed under CC BY 4.0

mRS, modified Rankin Scale

S2 Table. Nursing care levels and percentage of nursing care service users estimated according to modified Rankin Scale (mRS) score

|  | mRS | | | | | | |
| --- | --- | --- | --- | --- | --- | --- | --- |
|  | 0 | 1 | 2 | 3 | 4 | 5 | 6 |
| Nursing care level^21^ | No care | Support level 1 | Support level 2 | Care level 1 | Care level 2/3 | Care level 4/5 | NA |
| Percentage of nursing service users (%) [20,21] | 0 | 26.1 | 91.8 | 98.5 | 100 | 100 | 0 |

NA, not applicable

S3 Table. Results of deterministic sensitivity analysis from the public health payer’s perspective

| Parameters | Lower case | | | Base case | | | Upper case | | |
| --- | --- | --- | --- | --- | --- | --- | --- | --- | --- |
|  | SCT–control cost gap ($US) | ΔQALY | SCT cost ($US) | SCT–control cost gap ($US) | ΔQALY | SCT cost ($US) | SCT–control cost gap ($US) | ΔQALY | SCT cost ($US) |
| Risk ratio of the proportion of mRS scores of 0–2 | -23 | 0.00 | 151 | -565 | 0.10 | 3,746 | -1,590 | 0.28 | 10,531 |
| Medical cost | -453 | 0.10 | 3,633 |  |  |  | -679 | 0.10 | 3,859 |
| Utility | -565 | 0.10 | 3,675 |  |  |  | -565 | 0.10 | 3,818 |
| Percentage of mRS scores of 6 | -846 | 0.10 | 3,955 |  |  |  | -326 | 0.10 | 3,434 |
| Percentage of mRS scores of 0–2 in the control group | -290 | 0.05 | 1,921 |  |  |  | -1,424 | 0.28 | 10,234 |
| Discount rate | -565 | 0.11 | 3,990 |  |  |  | -565 | 0.09 | 3,532 |

mRS, modified Rankin Scale; QALY, quality-adjusted life year; SCT, stem cell therapy

S4 Table. Results of deterministic sensitivity analyses from the public healthcare and long-term care payer’s perspective

| Parameters | Lower case | | | Base case | | | Upper case | | | |
| --- | --- | --- | --- | --- | --- | --- | --- | --- | --- | --- |
|  | SCT–control cost gap ($US) | ΔQALY | SCT cost ($US) | SCT–control cost gap ($US) | ΔQALY | SCT cost ($US) | SCT–control cost gap ($US) | ΔQALY | SCT cost ($US) |  |
| Risk ratio of the proportion of mRS scores of 0–2 | -79 | 0.00 | 207 | -1,976 | 0.10 | 5,157 | -5,554 | 0.28 | 14,494 |  |
| Medical cost | - | 0.10 | 5,043 |  |  |  | -2,089 | 0.10 | 5,270 |  |
| Nursing care cost | -1,694 | 0.10 | 4,874 |  |  |  | -2,258 | 0.10 | 5,439 |  |
| Utility | -1,976 | 0.10 | 5,085 |  |  |  | -1,976 | 0.10 | 5,229 |  |
| Percentage of mRS scores of 6 | -2,256 | 0.10 | 5,437 |  |  |  | -1,736 | 0.10 | 4,916 |  |
| Percentage of mRS scores of 0–2 in the control group | -1,013 | 0.28 | 2,645 |  |  |  | -4,924 | 0.05 | 13,791 |  |
| Discount rate | -2,035 | 0.11 | 5,460 |  |  |  | -1,921 | 0.09 | 4,888 |  |

mRS, modified Rankin Scale; QALY, quality-adjusted life year; SCT, stem cell therapy


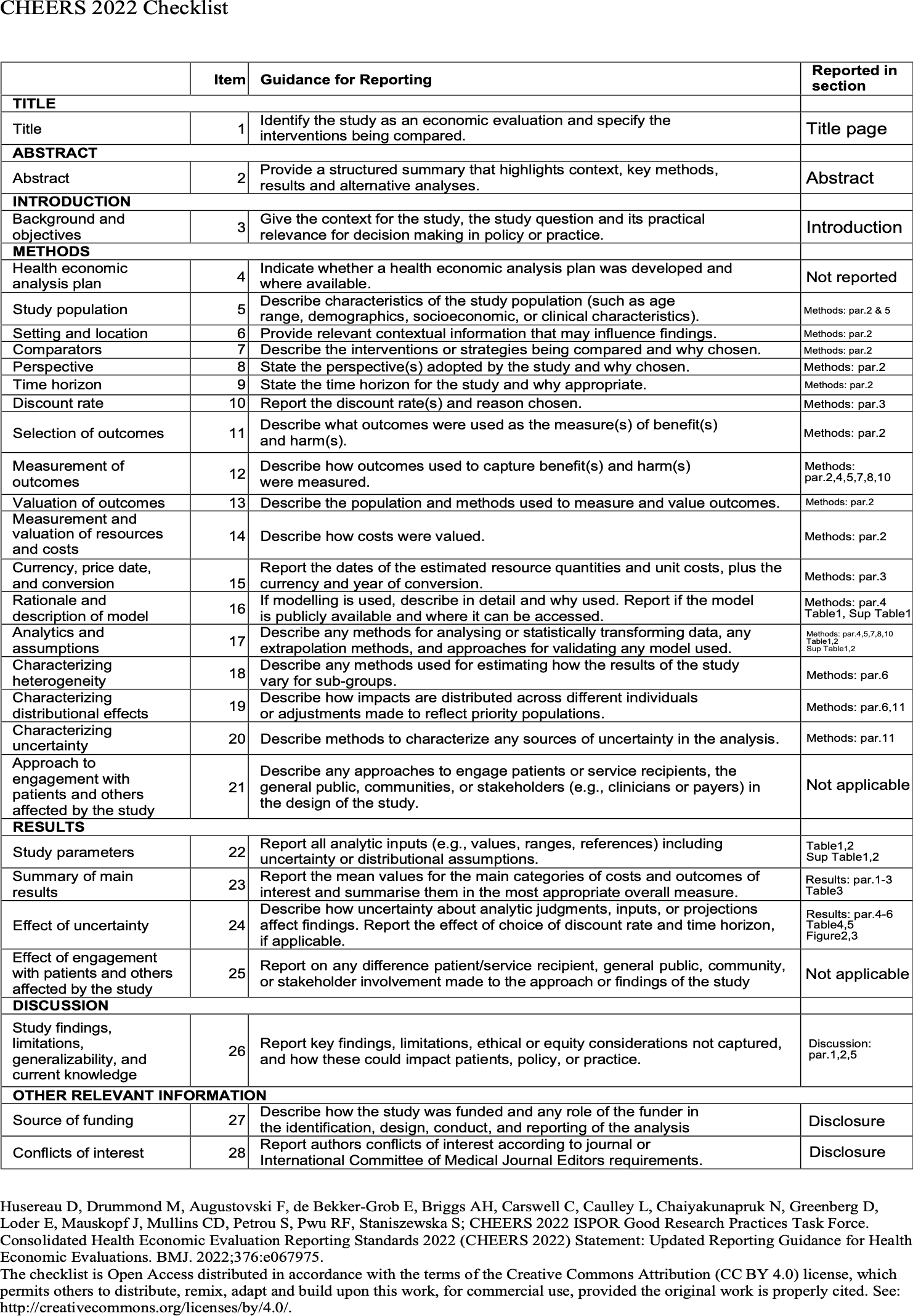


CHEERS 2022 Checklist

**Output data from the Markov model for the placebo arm**

|  | **mRS0-2** | **mRS3-5** | **mRS6** |
| --- | --- | --- | --- |
| **4 months** | 27.70% | 67.03% | 5.27% |
| **5 months** | 29.12% | 63.91% | 6.97% |
| **6 months** | 30.37% | 61.02% | 8.62% |
| **7 months** | 30.39% | 59.39% | 10.22% |
| **8 months** | 30.39% | 57.83% | 11.78% |
| **9 months** | 30.37% | 56.33% | 13.31% |
| **10 months** | 30.32% | 54.88% | 14.80% |
| **11 months** | 30.25% | 53.49% | 16.26% |
| **12 months** | 30.16% | 52.15% | 17.69% |
| **13 months** | 29.73% | 52.01% | 18.25% |
| **14 months** | 29.31% | 51.87% | 18.82% |
| **15 months** | 28.89% | 51.73% | 19.38% |
| **16 months** | 28.48% | 51.58% | 19.94% |
| **17 months** | 28.08% | 51.43% | 20.50% |
| **18 months** | 27.68% | 51.27% | 21.05% |
| **19 months** | 27.28% | 51.12% | 21.60% |
| **20 months** | 26.89% | 50.96% | 22.15% |
| **21 months** | 26.51% | 50.80% | 22.69% |
| **22 months** | 26.13% | 50.63% | 23.23% |
| **23 months** | 25.76% | 50.47% | 23.77% |
| **24 months** | 25.39% | 50.30% | 24.31% |
| **25 months** | 25.02% | 50.07% | 24.91% |
| **26 months** | 24.65% | 49.84% | 25.51% |
| **27 months** | 24.29% | 49.61% | 26.10% |
| **28 months** | 23.93% | 49.38% | 26.69% |
| **29 months** | 23.58% | 49.14% | 27.27% |
| **30 months** | 23.24% | 48.91% | 27.86% |
| **31 months** | 22.89% | 48.67% | 28.43% |
| **32 months** | 22.56% | 48.44% | 29.01% |
| **33 months** | 22.23% | 48.20% | 29.58% |
| **34 months** | 21.90% | 47.96% | 30.14% |
| **35 months** | 21.58% | 47.72% | 30.71% |
| **36 months** | 21.26% | 47.47% | 31.27% |
| **37 months** | 20.93% | 47.17% | 31.89% |
| **38 months** | 20.62% | 46.87% | 32.52% |
| **39 months** | 20.30% | 46.56% | 33.14% |
| **40 months** | 19.99% | 46.26% | 33.75% |
| **41 months** | 19.69% | 45.95% | 34.36% |
| **42 months** | 19.39% | 45.65% | 34.96% |
| **43 months** | 19.09% | 45.35% | 35.56% |
| **44 months** | 18.80% | 45.04% | 36.16% |
| **45 months** | 18.51% | 44.74% | 36.75% |
| **46 months** | 18.23% | 44.44% | 37.33% |
| **47 months** | 17.95% | 44.14% | 37.91% |
| **48 months** | 17.68% | 43.83% | 38.49% |
| **49 months** | 17.40% | 43.47% | 39.14% |
| **50 months** | 17.12% | 43.10% | 39.78% |
| **51 months** | 16.85% | 42.74% | 40.41% |
| **52 months** | 16.58% | 42.38% | 41.04% |
| **53 months** | 16.32% | 42.02% | 41.66% |
| **54 months** | 16.06% | 41.66% | 42.28% |
| **55 months** | 15.80% | 41.31% | 42.89% |
| **56 months** | 15.55% | 40.95% | 43.50% |
| **57 months** | 15.30% | 40.60% | 44.10% |
| **58 months** | 15.06% | 40.25% | 44.69% |
| **59 months** | 14.82% | 39.90% | 45.28% |
| **60 months** | 14.59% | 39.56% | 45.86% |
| **61 months** | 14.34% | 39.15% | 46.51% |
| **62 months** | 14.11% | 38.74% | 47.16% |
| **63 months** | 13.87% | 38.34% | 47.79% |
| **64 months** | 13.64% | 37.94% | 48.42% |
| **65 months** | 13.42% | 37.54% | 49.05% |
| **66 months** | 13.19% | 37.14% | 49.66% |
| **67 months** | 12.97% | 36.75% | 50.27% |
| **68 months** | 12.76% | 36.37% | 50.87% |
| **69 months** | 12.55% | 35.98% | 51.47% |
| **70 months** | 12.34% | 35.60% | 52.06% |
| **71 months** | 12.13% | 35.23% | 52.64% |
| **72 months** | 11.93% | 34.85% | 53.22% |
| **73 months** | 11.73% | 34.41% | 53.86% |
| **74 months** | 11.52% | 33.98% | 54.50% |
| **75 months** | 11.32% | 33.56% | 55.12% |
| **76 months** | 11.12% | 33.13% | 55.74% |
| **77 months** | 10.93% | 32.72% | 56.35% |
| **78 months** | 10.74% | 32.30% | 56.96% |
| **79 months** | 10.55% | 31.89% | 57.55% |
| **80 months** | 10.37% | 31.49% | 58.14% |
| **81 months** | 10.19% | 31.09% | 58.72% |
| **82 months** | 10.01% | 30.70% | 59.29% |
| **83 months** | 9.84% | 30.31% | 59.86% |
| **84 months** | 9.67% | 29.92% | 60.41% |
| **85 months** | 9.49% | 29.48% | 61.04% |
| **86 months** | 9.31% | 29.04% | 61.65% |
| **87 months** | 9.14% | 28.60% | 62.25% |
| **88 months** | 8.98% | 28.17% | 62.85% |
| **89 months** | 8.81% | 27.75% | 63.44% |
| **90 months** | 8.65% | 27.34% | 64.01% |
| **91 months** | 8.49% | 26.93% | 64.58% |
| **92 months** | 8.34% | 26.52% | 65.14% |
| **93 months** | 8.18% | 26.13% | 65.69% |
| **94 months** | 8.03% | 25.73% | 66.23% |
| **95 months** | 7.89% | 25.35% | 66.77% |
| **96 months** | 7.74% | 24.97% | 67.29% |
| **97 months** | 7.59% | 24.53% | 67.88% |
| **98 months** | 7.44% | 24.10% | 68.46% |
| **99 months** | 7.30% | 23.68% | 69.02% |
| **100 months** | 7.16% | 23.26% | 69.58% |
| **101 months** | 7.02% | 22.85% | 70.13% |
| **102 months** | 6.88% | 22.45% | 70.67% |
| **103 months** | 6.75% | 22.06% | 71.19% |
| **104 months** | 6.62% | 21.67% | 71.71% |
| **105 months** | 6.49% | 21.29% | 72.22% |
| **106 months** | 6.36% | 20.92% | 72.72% |
| **107 months** | 6.24% | 20.55% | 73.21% |
| **108 months** | 6.12% | 20.19% | 73.69% |
| **109 months** | 5.99% | 19.78% | 74.23% |
| **110 months** | 5.87% | 19.37% | 74.76% |
| **111 months** | 5.75% | 18.98% | 75.27% |
| **112 months** | 5.63% | 18.59% | 75.78% |
| **113 months** | 5.52% | 18.21% | 76.27% |
| **114 months** | 5.40% | 17.84% | 76.76% |
| **115 months** | 5.29% | 17.48% | 77.23% |
| **116 months** | 5.18% | 17.12% | 77.69% |
| **117 months** | 5.08% | 16.77% | 78.15% |
| **118 months** | 4.97% | 16.43% | 78.60% |
| **119 months** | 4.87% | 16.10% | 79.03% |
| **120 months** | 4.77% | 15.77% | 79.46% |

**Output data from the Markov model for the SCT arm**

|  | **mRS0-2** | **mRS3-5** | **mRS6** |
| --- | --- | --- | --- |
| **4 months** | 33.71% | 61.12% | 5.17% |
| **5 months** | 34.67% | 58.55% | 6.78% |
| **6 months** | 35.50% | 56.15% | 8.34% |
| **7 months** | 35.30% | 54.84% | 9.87% |
| **8 months** | 35.07% | 53.56% | 11.36% |
| **9 months** | 34.84% | 52.33% | 12.82% |
| **10 months** | 34.59% | 51.15% | 14.26% |
| **11 months** | 34.34% | 50.00% | 15.66% |
| **12 months** | 34.07% | 48.89% | 17.04% |
| **13 months** | 33.59% | 48.82% | 17.59% |
| **14 months** | 33.11% | 48.75% | 18.15% |
| **15 months** | 32.64% | 48.67% | 18.69% |
| **16 months** | 32.17% | 48.59% | 19.24% |
| **17 months** | 31.71% | 48.50% | 19.78% |
| **18 months** | 31.26% | 48.41% | 20.33% |
| **19 months** | 30.82% | 48.32% | 20.86% |
| **20 months** | 30.38% | 48.22% | 21.40% |
| **21 months** | 29.95% | 48.12% | 21.93% |
| **22 months** | 29.52% | 48.01% | 22.47% |
| **23 months** | 29.10% | 47.91% | 22.99% |
| **24 months** | 28.69% | 47.79% | 23.52% |
| **25 months** | 28.26% | 47.63% | 24.11% |
| **26 months** | 27.85% | 47.46% | 24.70% |
| **27 months** | 27.44% | 47.28% | 25.28% |
| **28 months** | 27.03% | 47.11% | 25.86% |
| **29 months** | 26.64% | 46.93% | 26.44% |
| **30 months** | 26.25% | 46.75% | 27.01% |
| **31 months** | 25.86% | 46.56% | 27.58% |
| **32 months** | 25.48% | 46.38% | 28.14% |
| **33 months** | 25.10% | 46.19% | 28.71% |
| **34 months** | 24.74% | 46.00% | 29.26% |
| **35 months** | 24.37% | 45.81% | 29.82% |
| **36 months** | 24.01% | 45.61% | 30.37% |
| **37 months** | 23.65% | 45.36% | 30.99% |
| **38 months** | 23.29% | 45.10% | 31.61% |
| **39 months** | 22.93% | 44.85% | 32.22% |
| **40 months** | 22.58% | 44.59% | 32.83% |
| **41 months** | 22.24% | 44.33% | 33.43% |
| **42 months** | 21.90% | 44.07% | 34.03% |
| **43 months** | 21.56% | 43.81% | 34.62% |
| **44 months** | 21.23% | 43.55% | 35.21% |
| **45 months** | 20.91% | 43.29% | 35.80% |
| **46 months** | 20.59% | 43.03% | 36.38% |
| **47 months** | 20.28% | 42.77% | 36.95% |
| **48 months** | 19.97% | 42.51% | 37.53% |
| **49 months** | 19.65% | 42.18% | 38.17% |
| **50 months** | 19.34% | 41.86% | 38.81% |
| **51 months** | 19.03% | 41.53% | 39.44% |
| **52 months** | 18.73% | 41.21% | 40.06% |
| **53 months** | 18.43% | 40.89% | 40.68% |
| **54 months** | 18.14% | 40.57% | 41.30% |
| **55 months** | 17.85% | 40.25% | 41.90% |
| **56 months** | 17.57% | 39.93% | 42.51% |
| **57 months** | 17.29% | 39.61% | 43.10% |
| **58 months** | 17.01% | 39.29% | 43.69% |
| **59 months** | 16.74% | 38.98% | 44.28% |
| **60 months** | 16.48% | 38.66% | 44.86% |
| **61 months** | 16.20% | 38.28% | 45.51% |
| **62 months** | 15.93% | 37.91% | 46.16% |
| **63 months** | 15.67% | 37.54% | 46.79% |
| **64 months** | 15.41% | 37.17% | 47.42% |
| **65 months** | 15.15% | 36.80% | 48.05% |
| **66 months** | 14.90% | 36.44% | 48.66% |
| **67 months** | 14.66% | 36.07% | 49.27% |
| **68 months** | 14.41% | 35.71% | 49.87% |
| **69 months** | 14.17% | 35.36% | 50.47% |
| **70 months** | 13.94% | 35.00% | 51.06% |
| **71 months** | 13.71% | 34.65% | 51.64% |
| **72 months** | 13.48% | 34.30% | 52.22% |
| **73 months** | 13.24% | 33.89% | 52.87% |
| **74 months** | 13.01% | 33.48% | 53.51% |
| **75 months** | 12.79% | 33.08% | 54.14% |
| **76 months** | 12.56% | 32.68% | 54.76% |
| **77 months** | 12.35% | 32.28% | 55.37% |
| **78 months** | 12.13% | 31.89% | 55.98% |
| **79 months** | 11.92% | 31.50% | 56.58% |
| **80 months** | 11.71% | 31.12% | 57.17% |
| **81 months** | 11.51% | 30.74% | 57.75% |
| **82 months** | 11.31% | 30.37% | 58.32% |
| **83 months** | 11.11% | 30.00% | 58.89% |
| **84 months** | 10.92% | 29.63% | 59.45% |
| **85 months** | 10.72% | 29.20% | 60.08% |
| **86 months** | 10.52% | 28.78% | 60.70% |
| **87 months** | 10.33% | 28.36% | 61.31% |
| **88 months** | 10.14% | 27.95% | 61.91% |
| **89 months** | 9.95% | 27.55% | 62.50% |
| **90 months** | 9.77% | 27.15% | 63.08% |
| **91 months** | 9.59% | 26.75% | 63.65% |
| **92 months** | 9.42% | 26.36% | 64.22% |
| **93 months** | 9.24% | 25.98% | 64.78% |
| **94 months** | 9.07% | 25.60% | 65.32% |
| **95 months** | 8.91% | 25.23% | 65.86% |
| **96 months** | 8.74% | 24.86% | 66.40% |
| **97 months** | 8.57% | 24.44% | 66.99% |
| **98 months** | 8.41% | 24.02% | 67.57% |
| **99 months** | 8.25% | 23.61% | 68.15% |
| **100 months** | 8.09% | 23.20% | 68.71% |
| **101 months** | 7.93% | 22.81% | 69.26% |
| **102 months** | 7.77% | 22.42% | 69.81% |
| **103 months** | 7.62% | 22.03% | 70.34% |
| **104 months** | 7.48% | 21.65% | 70.87% |
| **105 months** | 7.33% | 21.28% | 71.39% |
| **106 months** | 7.19% | 20.92% | 71.89% |
| **107 months** | 7.05% | 20.56% | 72.39% |
| **108 months** | 6.91% | 20.21% | 72.88% |
| **109 months** | 6.77% | 19.80% | 73.43% |
| **110 months** | 6.63% | 19.41% | 73.96% |
| **111 months** | 6.49% | 19.02% | 74.48% |
| **112 months** | 6.36% | 18.64% | 75.00% |
| **113 months** | 6.23% | 18.27% | 75.50% |
| **114 months** | 6.10% | 17.90% | 75.99% |
| **115 months** | 5.98% | 17.55% | 76.48% |
| **116 months** | 5.85% | 17.20% | 76.95% |
| **117 months** | 5.73% | 16.85% | 77.41% |
| **118 months** | 5.62% | 16.52% | 77.87% |
| **119 months** | 5.50% | 16.19% | 78.31% |
| **120 months** | 5.39% | 15.86% | 78.75% |
