# Supplementary material for: Cost-effectiveness of stem cell therapy versus standard of care for acute and subacute ischemic stroke
Source: PLoS One. 2026 May 21;21(5):e0349756. doi: 10.1371/journal.pone.0349756 (PMC13193451; doi:10.1371/journal.pone.0349756)
Supplement: Attachment — Submitted filename: Response_v3.docx [file pone.0349756.s003.docx]

０Journal Requirements:

When submitting your revision, we need you to address these additional requirements

1. Please ensure that your manuscript meets PLOS ONE's style requirements, including those for file naming. The PLOS ONE style templates can be found at

https://journals.plos.org/plosone/s/file?id=wjVg/PLOSOne_formatting_sample_main_body.pdf and

https://journals.plos.org/plosone/s/file?id=ba62/PLOSOne_formatting_sample_title_authors_affiliations.pdf.

Response： We sincerely appreciate your helpful guidance. The manuscript and all associated files have been thoroughly revised to ensure full compliance with PLOS ONE’s formatting and file naming requirements, following the provided templates.

2. Please note that PLOS One has specific guidelines on code sharing for submissions in which author-generated code underpins the findings in the manuscript. In these cases, we expect all author-generated code to be made available without restrictions upon publication of the work. Please review our guidelines at https://journals.plos.org/plosone/s/materials-and-software-sharing#loc-sharing-code and ensure that your code is shared in a way that follows best practice and facilitates reproducibility and reuse.

Response：Thank you for your reminder and for providing the detailed guidelines on code sharing. Our R codes for the cost-utility analysis will be available upon reasonable requests.

3. If the reviewer comments include a recommendation to cite specific previously published works, please review and evaluate these publications to determine whether they are relevant and should be cited. There is no requirement to cite these works unless the editor has indicated otherwise.

Response：The reviewers did not mention any specific published works.

Reviewers' comments:

Reviewer's Responses to Questions

Comments to the Author

1. Is the manuscript technically sound, and do the data support the conclusions?

The manuscript must describe a technically sound piece of scientific research with data that supports the conclusions. Experiments must have been conducted rigorously, with appropriate controls, replication, and sample sizes. The conclusions must be drawn appropriately based on the data presented.

Reviewer #1: Yes

Reviewer #2: Yes

2. Has the statistical analysis been performed appropriately and rigorously?

Reviewer #1: Yes

Reviewer #2: I Don't Know

3. Have the authors made all data underlying the findings in their manuscript fully available?

The PLOS Data policy requires authors to make all data underlying the findings described in their manuscript fully available without restriction, with rare exception (please refer to the Data Availability Statement in the manuscript PDF file). The data should be provided as part of the manuscript or its supporting information, or deposited to a public repository. For example, in addition to summary statistics, the data points behind means, medians and variance measures should be available. If there are restrictions on publicly sharing data—e.g. participant privacy or use of data from a third party—those must be specified.

Reviewer #1: Yes

Reviewer #2: Yes

4. Is the manuscript presented in an intelligible fashion and written in standard English?

PLOS ONE does not copyedit accepted manuscripts, so the language in submitted articles must be clear, correct, and unambiguous. Any typographical or grammatical errors should be corrected at revision, so please note any specific errors here.

Reviewer #1: Yes

Reviewer #2: Yes

5. Review Comments to the Author

Please use the space provided to explain your answers to the questions above. You may also include additional comments for the author, including concerns about dual publication, research ethics, or publication ethics. (Please upload your review as an attachment if it exceeds 20,000 characters)

Reviewer #1: Takamiya and Morii et al. demonstrated the range of SCT costs at which the cost- effectiveness of SCT for patients with acute and subacute ischemic stroke becomes superior to that of SOC treatment alone. I believe this is a valuable study in terms of clarifying the importance of stem cell treatment in a statistical manner. Moreover, I agree that choosing the medical system in Japan to estimate costs for the cost-effectiveness analysis is a good idea since Japanese health care system is being challenged with aging population. However, my opinion is that this study should be easily read by patients, their relatives, and the law makers (since it has crucial analyses for stem cell treatment) in addition to our scientific community. In this manner, I highly recommend the authors to explain biostatistical terms in more detail in this paper where it is possible to make this paper more easily understandable.

I am hoping that my recommendations below will improve this paper.

-Table 1. 1) Please clearly explain in the table title that what are these percentages.

Response：Thank you. We have revised the table title as “ Proportion of mRS stages at 3 months in the Markov model derived from the meta-analysis by Osanai et al.^[10]^”

2) Please add patient numbers for mRS score 3-5 in the base, scenario 1 and 2.

Response：Thank you for pointing this out. In this study, the proportion of patients with mRS 3–5 (patients with disability) was calculated as a residual value by subtracting the proportions of patients with functional independence (mRS 0–2) and death (mRS 6) from 100%. Therefore, the absolute patient numbers for mRS 3–5 cannot be directly derived from the available data. We agree that the inclusion of patient numbers for mRS 0–2 and 6 in Table 1 may be confusing, and we are willing to remove these numbers from Table 1 if deemed appropriate by the editor and reviewers.

3) Please add short explanations on what the base, scenarios 1 and 2 are on table 1 so that everybody can see what they stand for on the table quickly.

Response：Thank you very much for your suggestion. We have added following explanations to Table 1 to clarify the definitions of the base case and each scenario, so that readers can easily understand their meaning; Base case (all studies in the meta-analysis), Scenario analysis 1 (studies with SCT ≤30 days of stroke onset), and Scenario analysis 2 (studies conducted in Japan).

-Page 6 Line 130-135: too many uses of “that study” in writing. Please rewrite this paragraph so that it could be clear for everybody.

Response：Thank you for your valuable suggestion. We agree that the repetitive use of 'that study' was potentially confusing. We have revised the paragraph to clarify the distinction between the current study and the referenced meta-analysis. Specifically, we replaced 'that study' with 'the meta-analysis' and 'that work' to improve readability and precision.

-Page 7 Line 156. Do not assume that only biostatisticians are going to read your paper and please explain what you mean with “Initial Probabilities in Table 1” for the people with little or no statistics knowledge. Your paper should be readable for general public, as well, since the topic of your paper is very important to the patients, their relatives, and policy makers to make a decision in these treatments.

Response：We sincerely appreciate this comment. We recognize the importance of making our findings accessible to a broader audience, including patients and policy makers. Accordingly, we have revised the text to define 'initial probabilities' as the starting health status of patients following treatment. We have also added a brief explanation of how these probabilities represent the distribution of functional outcomes, ensuring the baseline of our model is clear to non-statisticians.

-Table 2 is very unorganized in this current format. Please make sure that your table has a decent look. You may add fill all borders with lines so that we can see the columns and rows of the table clearly.

Response：Thank you for your feedback. To enhance the visibility of Table 2, we have added borders to all cells. In the revised table, each row and column can now be clearly distinguished.

Again, please explain what beta, gama, and care levels are in the title of the table.

Response：Thank you for your comment. We have added description on what the beta and gamma mean and additional explanation on the sensitivity analysis in the “Sensitivity analyses” section.

-Page 10 Line 179: How did you decide nursing support and care levels? If there is a published standard, please cite. If not, please explain

Response：We had described that in our previous manuscript (Line 181-188 in our previous manuscript). The correspondence of mRS scores to nursing care levels were on Table S2. This is in accordance with previous studies. We have added reference information on the manuscript.

-Similar to Table 2, Table 3 is also unorganized. Please read my comment for Table 2 above and make rows and columns easily distinguishable.

Response：Thank you very much for your feedback. In response to your comments on Table 2, we have similarly revised the format of Table 3. In addition, we have applied the same adjustments to Table 1, including the addition of borders and layout reorganization. This ensures consistent readability across all tables.

-Figures 2 and 3: I recommend adding the percentage and the risk ratio of mRS 3-5 in figures 2 and 3 in the sensitivity analysis for the sake of being more informative in these graphs since there is a risk that the disease status of the patients might change from 0-2 to 3-5.

Response：Thank you for your suggestion. We think the sensitivity analysis should be without the parameter mRS3-5. In the upper side of Figure 3, the impact of changes in parameters such as percentage of mRS0–2 in the control group. In this study, the proportion of mRS3-5 is defined as the complement of mRS0-2 and mRS6 (equal percentage between groups). Therefore, the effect of mRS0-2 and mRS3-5 are fundamentally the same because if the percentage of mRS0-2 increases by 1%, that of mRS3-5 decreases by 1%, and vice versa. Also, our meta-analysis show that the proportion of mRS0-2, not mRS3-5, increases by stem cell therapy. Therefore, it is appropriate that the impact of the proportion of and the risk ratio of mRS0-2 in the sensitivity analysis.

Discussion:

Page 16 line 288: Discuss what clinical trial demonstrated in more detail.

Response: Thank you for this insightful suggestion. We have revised the Discussion section to provide more specific clinical details from the RCTs used in our meta-analysis. Specifically, we have added information regarding the administration routes, the specific patient population (acute/subacute), and the key efficacy and safety results—such as the 32.6% vs. 26.1% achievement rate of mRS 0–2 and the 3.5% mortality rate. We believe these additions clarify the clinical foundation of our cost-effectiveness model ( Lines 335- 346 in the revised manuscript).

Conclusions:

Your conclusion is missing your scenarios. Please add them in your conclusion as well and tell us what the importance of those scenarios is in addition to mentioning threshold costs only for base-case scenario. Have a more comprehensive and inclusive conclusion.

Response: We appreciate the reviewer’s valuable suggestion. We have significantly revised the Conclusion section to include the results in our scenario analyses. We have also added a discussion on the importance of these scenarios, emphasizing how treatment timing and regional data alignment influence the economic value of SCT. This provides a more comprehensive perspective on the therapy's cost-effectiveness.

-Author contributions are missing. Please clearly state who did what job in this paper.

Response：Thank you for the comment. While the PLOS ONE format typically does not require author contributions in a manuscript, we have added an "Author Contributions" section at the end of the manuscript in response to your suggestion. We leave the final decision regarding its inclusion in the final text to the Editor's discretion.

Reviewer #2: Takamiya et al. have performed a cost-effectiveness analysis of stem cell therapy when compared to standard of care in the treatment of ischemic stroke. They have presented their findings in the context of the Japanese healthcare system and provide a set of threshold costs that would make stem cell therapy cost-effective for application in the treatment of acute/sub-acute ischemic stroke. While the study is interesting as it focuses on the financial implications of stem cell therapy in a condition that may see increased application in the near future, certain aspects of the manuscript require further clarification to make it easier to interpret for the readers.

Comments:

1. While the abstract is overall well-written, it is unclear when the authors refer to the meta-analysis that has been the basis of this manuscript. The authors should better present/explain this in the abstract.

Response: Thank you for your insightful comment. We have revised the abstract to clarify the relationship between our study and the meta-analysis mentioned.

2. In the introduction, there is sufficient focus on the economic perspective of stroke treatment. It will also be nice to include the biological basis of stem cell therapy for ischemic stroke, to put it into perspective for the readers why this comparison is being made with the standard of care. The standard of care should also be briefly described along with this (acute reperfusion therapies, line 52).

Response: Thank you for your constructive suggestion. Following your advice, we have revised the Introduction to include the biological basis of stem cell therapy, highlighting its mechanisms such as neuroprotection and immunomodulation. We have also provided a brief description of the current standard of care, specifically mentioning acute reperfusion therapies (intravenous thrombolysis and mechanical thrombectomy) to clarify the clinical context and the rationale for our comparison.

3. In line 58, please include the statistics as done in line 59.

Response: Thank you for your comment. We apologize for the lack of clarity in the previous version of the manuscript. Given that this analysis is based on the risk ratio between the SCT and control groups, we have revised the text to present the frequency data for both the SCT group (32.6%) and the control group (26.1%) alongside the comparative statistics to facilitate a better understanding for the reader.

4. In line 66, please elaborate on the statement that the “assumption is unrealistic”. Please cite suitable references in support of your statement(s).

Response: Thank you for your comment. We have revised the Introduction to clarify why a functional plateau at 3 months is considered unrealistic and have added a supporting reference.

A longitudinal study by Lee et al. (2015) demonstrated that neurological and functional impairments—including ADL and gait—continue to improve significantly for up to 6 months post-stroke. Their multi-time-point analysis explicitly showed that recovery variables had not yet reached a plateau between 3 and 6 months. Additionally, other cost-effectiveness studies (references 24, 27) have examined long-term changes in mRS. Therefore, we used a Markov model to more accurately reflect these ongoing clinical transitions compared to a static model. We have also revised the term "unrealistic" to "inappropriate" to moderate the strength of the expression.

5. At the end of the introduction, please clearly highlight the hypothesis and research questions being addressed by this study.

Response: Thank you for your suggestion. We have revised the final paragraph of the Introduction to explicitly state our research question and hypothesis. Specifically, we added that this study aims to investigate the cost-effectiveness of SCT from the Japanese public payer's perspective, testing the hypothesis that SCT is cost-effective within certain price thresholds. We believe these additions clarify the objectives of our study while maintaining the original context.

6. In line 79, there is an extra period symbol.

Response：Thank you for pointing this out. We have corrected the typographical error by removing the extra period in line 79 in the previous manuscript.

7. In line 86 and 91, the authors introduce the terminology of “public health payer’s perspective” and “public healthcare and long-term care payer”, respectively. Please elaborate on these terms so that it is also clear for a reader to interpret this who do not have a background in this field.

Response：Thank you for your comment. We have added description on the 2 perspectives in the “Participants and setting”. We hope this revision will increase readability for readers.

8. In line 107, the authors state that they use the currency rate as per December 2024. It should be relatively easy to adjust the conversion rates to a more recent period in 2025, to make the comparison more relevant.

Response：Thank you. We have revised the results using the latest currency rate.

9. In line 132, the authors state the dataset used in the main analysis of another study. Please describe this dataset in brief, to put it in context for the readers of the current manuscript.

Response: Thank you for this helpful suggestion. Following your advice, we have added a brief description of the dataset from our previous meta-analysis (reference [10]) in the Methods section. We have clarified the number of randomized controlled trials included, the total number of patients, and the primary focus of those studies (e.g., mesenchymal stem cell transplantation for acute/subacute stroke). This addition helps to provide necessary context for the clinical data used in our current cost-effectiveness simulation.

10. In line 134, the authors state that there was a significant difference in the comparison. Please add the statistics to support this.

Response: Thank you for your suggestion. We have added the specific statistical results from the meta-analysis—including the risk ratio , 95% confidence interval, p-value, and I2 statistic—to the manuscript to support the statement of significant difference.

11. In line 138 and 144, please link the supplementary material accordingly as it is unclear that this has been highlighted there.

Response: Thank you for your comment. We have revised the manuscript to clearly specify the locations of the supplementary information for both scenarios. For Scenario 1, we added a direct reference to Figure S2 in the supplementary material of our previous study. For Scenario 2, we have provided the methodological details and the resulting forest plot in the Supplementary Material of the current manuscript. These updates ensure that the evidence for our analysis is transparent and easily accessible.

12. Please add references for the statement made in line 141 and briefly elaborate.

Response：Thank you for your suggestion. Following your advice, we have added appropriate references in line 162 in the revised manuscript.

13. I would recommend that the authors move the methods and results text from the supplementary materials to the main manuscript. This will increase clarity of the manuscript. Please only include figures and tables in the supplementary material.

Response：Thank you for this constructive recommendation. We agree that moving the descriptive text to the main manuscript significantly improves clarity. As requested, the Supplementary Material now consists solely of figures and tables (S1–S4 Figs and S1–S4 Tables).

14. Please briefly elaborate on the statement in line 173 and 174 with suitable references.

Response：Thank you for this important comment. In response, we have added the following sentence to the relevant section:

"Since there has been no evidence that long-term outcomes such as recurrence rates differ between the groups, medical costs related to recurrence or long-term follow-up were considered the same and not included in the analysis."

15. Please provide suitable references for the statements in lines 181 – 184.

Response：Thank you for your valuable suggestion. We have added a reference from the Ministry of Health, Labour and Welfare (MHLW) of Japan to support the description of the nursing care insurance system and the certification process for nursing care levels (Lines 234 in the revised manuscript). The reference has been added to the manuscript as Reference [13,15,16].

16. In line 195, please explain in brief about the base-case analysis used in this manuscript.

Response：Thank you for your comment. The definition and settings of the base-case analysis are described in the Methods section (Lines 148–150 in the revised manuscript). Following your suggestion, we have added a brief clarifying phrase and a cross-reference to Line 243-245 in the revised manuscript to remind the reader of the analysis framework without being repetitive.

17. Please expand the acronym QALYs in the methods section header (line 198).

Response：We have expanded the acronym "QALYs" to "Quality-Adjusted Life Years" in the header of the Methods section (Line 247 in the revised manuscript)

18. In the results section, I would request the authors to provide a “take home message” and the end of every result subsection that briefly describes the findings that they report. This will increase readability of the results.

Response：Thank you for your constructive suggestion to improve the readability of our manuscript. While we appreciate the importance of clarity, we have followed the standard IMRAD (Introduction, Methods, Results, and Discussion) format, where the Results section is dedicated to the objective presentation of data without interpretation. To maintain the scientific objectivity of this section and avoid redundancy, we have ensured that the key findings (take-home messages) of each subsection are clearly highlighted and synthesized in the first paragraph of the Discussion section. We believe this structure aligns with general editorial practices and provides the clarity you suggested.

19. In figures 2 and 3, also state the reported values for each variable in the tornado plot. This will increase the readability of the plots.

Response：Thank you for this helpful suggestion. As recommended, we have added the reported values for each variable to the tornado plots in Figures 2 and 3 to improve readability.

20. In the discussion, I would urge the authors to put into perspective the reported cost-effectiveness analysis for stem cell therapy in ischemic stroke with other cell based therapies. This will make it easier for the reader the interpret the reported financial aspects better.

Response：Thank you for this valuable suggestion. We agree that comparing our results with other cell treatments makes the costs easier to understand. As suggested, we have added a paragraph to the Discussion section comparing the threshold costs of SCT for ischemic stroke with those of autologous bone‑marrow–derived mesenchymal stem cell (STEMIRAC ®)therapy for traumatic spinal cord injury. We highlighted the difference in pricing considerations between diseases with no effective treatment available and common diseases like stroke, which carry a substantial long-term care burden and require sustainable pricing strategies.

21. I would also ask the authors to have a discussion of their findings from Japan in the context of other major healthcare systems from the UK-EU, US-Canada and major Asian economies (such as India, Singapore, China). Can these findings be extrapolated to other regions? Would any suitable adjustments be required to be made based on socio-economic features?

Response: Thank you for this insightful comment regarding the generalizability of our findings. We agree that socio-economic features and the structure of healthcare systems are critical factors in cost-effectiveness. However, we believe that a detailed cross-country comparison is challenging for the following reasons:

1. Complexity and Regional Specificity: National healthcare systems, including reimbursement structures, insurance models, and Willingness-to-Pay (WTP) thresholds, are profoundly complex and uniquely structured for each region. Accurate extrapolation would require deep, localized expertise in each specific healthcare economy to avoid misleading conclusions.
2. Lack of Approved Therapies: To the best of our knowledge, no cell-based therapy for ischemic stroke has yet been formally approved or assigned a market price in the regions mentioned by the reviewer. Without these real-world benchmarks, any comparative financial analysis would remain largely speculative.

In the revised manuscript, we have added a brief statement in the Discussion section noting these regional limitations and emphasizing that while our Japanese dataset provides a foundational model, direct application to other regions should be interpreted with caution (in line 373-379 in the revised manuscript).

22. The authors should also discuss their choice of using a Markov model for the analysis in this manuscript. This may also be done in the discussion section. Please also put into context if similar statistical approaches have been used in prior studies that evaluate cost-effectiveness of different treatment groups. This may be done in either in the methods or discussion section.

Response：Thank you for your suggestion. Markov modeling is a commonly used modeling method for cost-effectiveness analysis. Markov model is commonly used for chronic disease because the model simulate patients’ health states over time at a regular cycle. The modeling is also commonly used for stroke after post-acute stages. We have added some description in the method section(“Markov model” section) and added some references.

23. Can the authors please add the output data from running the Markov models in the supplementary material? This will make it easier for the readers to follow and interpret the reported results.

Response：Thank you. We have added the output from the Markov model on the distributions of mRS scores over time as a supplementary material.

6. PLOS authors have the option to publish the peer review history of their article (what does this mean?). If published, this will include your full peer review and any attached files.

If you choose “no”, your identity will remain anonymous but your review may still be made public.

Do you want your identity to be public for this peer review? For information about this choice, including consent withdrawal, please see our Privacy Policy.

Reviewer #1: No

Reviewer #2: No
