# Supplementary material for: Cost-effectiveness of stem cell therapy versus standard of care for acute and subacute ischemic stroke
Source: PLoS One. 2026 May 21;21(5):e0349756. doi: 10.1371/journal.pone.0349756 (PMC13193451; doi:10.1371/journal.pone.0349756)
Supplement: Attachment — Submitted filename: Response_v4.docx [file pone.0349756.s004.docx]

０Journal Requirements:

If the reviewer comments include a recommendation to cite specific previously published works, please review and evaluate these publications to determine whether they are relevant and should be cited. There is no requirement to cite these works unless the editor has indicated otherwise. 

Response: No specific publications were recommended for citation by the reviewers. Accordingly, no additional references have been added on this basis.

Please review your reference list to ensure that it is complete and correct. If you have cited papers that have been retracted, please include the rationale for doing so in the manuscript text, or remove these references and replace them with relevant current references. Any changes to the reference list should be mentioned in the rebuttal letter that accompanies your revised manuscript. If you need to cite a retracted article, indicate the article’s retracted status in the References list and also include a citation and full reference for the retraction notice.

Response: We have reviewed our reference list and confirmed that none of the cited works have been retracted. No changes to the reference list were necessary.

Reviewers' comments:

Reviewer's Responses to Questions

Comments to the Author

1. If the authors have adequately addressed your comments raised in a previous round of review and you feel that this manuscript is now acceptable for publication, you may indicate that here to bypass the “Comments to the Author” section, enter your conflict of interest statement in the “Confidential to Editor” section, and submit your "Accept" recommendation.

Reviewer #1: All comments have been addressed

Reviewer #2: All comments have been addressed

2. Is the manuscript technically sound, and do the data support the conclusions?

The manuscript must describe a technically sound piece of scientific research with data that supports the conclusions. Experiments must have been conducted rigorously, with appropriate controls, replication, and sample sizes. The conclusions must be drawn appropriately based on the data presented.

Reviewer #1: Yes

Reviewer #2: Yes

3. Has the statistical analysis been performed appropriately and rigorously?

Reviewer #1: Yes

Reviewer #2: Yes

4. Have the authors made all data underlying the findings in their manuscript fully available?

The PLOS Data policy requires authors to make all data underlying the findings described in their manuscript fully available without restriction, with rare exception (please refer to the Data Availability Statement in the manuscript PDF file). The data should be provided as part of the manuscript or its supporting information, or deposited to a public repository. For example, in addition to summary statistics, the data points behind means, medians and variance measures should be available. If there are restrictions on publicly sharing data—e.g. participant privacy or use of data from a third party—those must be specified.

Reviewer #1: Yes

Reviewer #2: Yes

5. Is the manuscript presented in an intelligible fashion and written in standard English?

PLOS ONE does not copyedit accepted manuscripts, so the language in submitted articles must be clear, correct, and unambiguous. Any typographical or grammatical errors should be corrected at revision, so please note any specific errors here.

Reviewer #1: Yes

Reviewer #2: Yes

6. Review Comments to the Author

Please use the space provided to explain your answers to the questions above. You may also include additional comments for the author, including concerns about dual publication, research ethics, or publication ethics. (Please upload your review as an attachment if it exceeds 20,000 characters)

Reviewer #1: Thanks so much for addressing my points. I request that you address my last point below:

Table 1. Please explain under the table (along with the abbreviations) why you could not include direct patient numbers for mRS score 3-5 rather than deleting patient numbers for other mRS groups.

Response: Thank you for this suggestion. As recommended, we have added the following footnote to Table 1 to clarify why absolute patient numbers for mRS 3–5 could not be provided:

"The proportion of patients with mRS 3–5 (disability) was calculated as a residual by subtracting the proportions of mRS 0–2 (functional independence) and mRS 6 (death) from 100%; therefore, absolute patient numbers for mRS 3–5 are not directly available."

Reviewer #2: I thank the authors for revising the manuscript on the basis of my review. I find the manuscript to be acceptable for publication in its current form.

Response: We sincerely thank the reviewer for the thoughtful evaluation and constructive comments, which have helped improve the quality of our manuscript.

7. PLOS authors have the option to publish the peer review history of their article (what does this mean?). If published, this will include your full peer review and any attached files.

If you choose “no”, your identity will remain anonymous but your review may still be made public.

Do you want your identity to be public for this peer review? For information about this choice, including consent withdrawal, please see our Privacy Policy.

Reviewer #1: No

Reviewer #2: No
